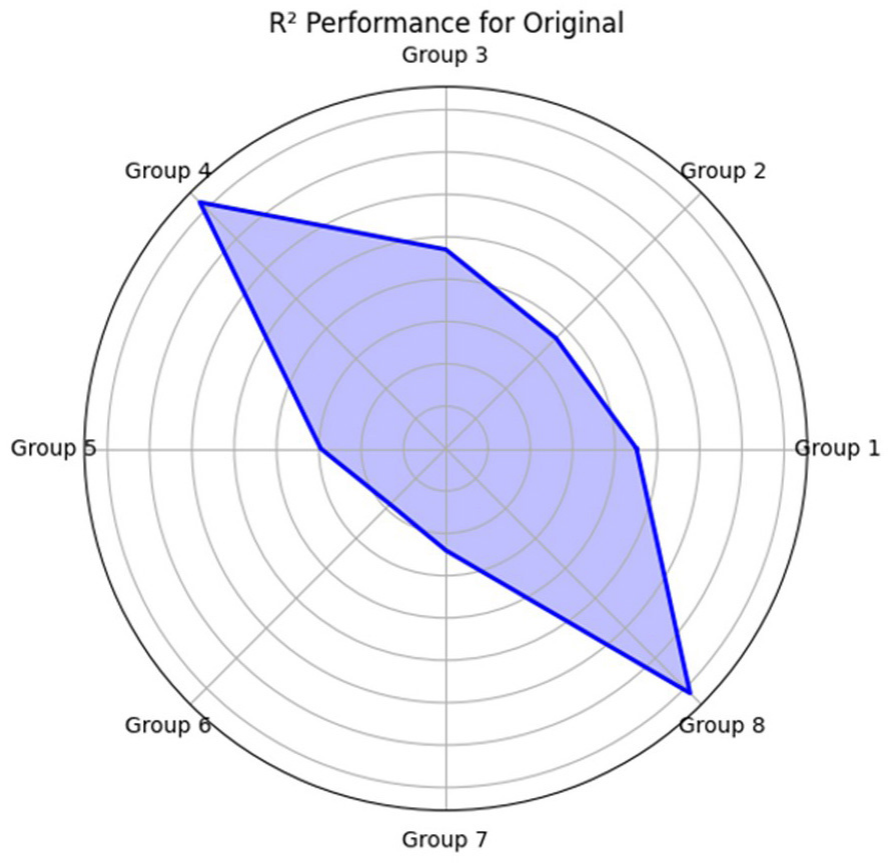# 59 Length of Stay Prediction Enhanced by Admission AM-PAC 6-Clicks Score

**DOI:** 10.1093/jbcr/iraf019.059

**Published:** 2025-04-01

**Authors:** Hannah Moore, Douglas Bettarelli, Vishal Bandaru, Kurt Grabow, Khaja Siddiqui, Mark Gao, Rafael Cacao, Senja Collins, Chip Shaw, Alan Pang, John Griswold

**Affiliations:** Burn Center at University Medical Center; Texas Tech University Health Sciences Center; Texas Tech University Health Sciences Center; Texas Tech University Health Sciences Center School of Medicine; Texas Tech University Health Sciences Center School of Medicine; Texas Tech University Health Sciences Center; Texas Tech University; Trustpoint Rehabilitation Hospital of Lubbock; Texas Tech University Health Sciences Center; Texas Tech University Health Sciences Center School of Medicine; Texas Tech University Health Sciences Center School of Medicine

## Abstract

**Introduction:**

Burn injuries are some of the most severe injuries that enter hospitals. Length of stay (LOS) is often difficult to predict in these patients, and the variability in individual responses to care makes it difficult to estimate burn recovery. Burn mortality prediction is well-established, but as survival rates rapidly increase, formulas have to be readjusted. Physical therapists gauge mobility with a tool known as AM-PAC 6-clicks on a scale of 6 to 24, with lower scores implying worse mobility. These tools offer an interprofessional perspective on predicting LOS outcomes.

**Methods:**

Data was collected from 211 burn patients, aged 18 - 89, admitted between January 1, 2021, and May 31, 2023. Mobility was assessed using the 6-clicks score upon admission, alongside TBSA, age, gender, number of surgeries, LOS, and intubation status. A multiple linear regression was performed using 8 different groups of features to predict LOS. Groups 1 to 4 included 6-clicks score, while groups 5 to 8 were created without it. Groups 1 and 4 evaluated using TBSA, groups 2 and 5 evaluated using 2nd degree and 3rd degree burns and total TBSA separately, groups 3 and 6 added ages, and intubation, groups 4 and 8 added number of inpatient surgeries. The performance of the models were evaluated using Mean Squared Error (MSE) and R² metrics. The models were trained on 80% of the data and tested on the remaining 20%.

**Results:**

The 6-clicks score on admission consistently emerged as a significant predictor of LOS in burn patients across all models, with a strong negative correlation with LOS. In Group 1 (B = -0.810, p < 0.001, CI [-1.225, -0.394]), where TBSA and LOS were the only predictors, and (B = -0.432, p = 0.004, CI [-0.726, -0.138]), where all the features were included, the 6-clicks score retained its significance even with the additional clinical factors. In comparison, models excluding the score, like Group 8, had reduced predictive capability. These results highlight the 6-clicks score as a key factor in predicting LOS, reinforcing its value in clinical models.

**Conclusions:**

The inclusion of the 6-clicks score enhanced the accuracy of the length of stay predictions across the entire dataset. Groups 1 and 4 showed that combining 6-clicks with total TBSA provided better predictions than using TBSA alone. Notably, Group 4, which included 6-clicks, resulted in the most reliable prediction model.

**Applicability of Research to Practice:**

Interprofessional efforts offer new insights into predictive data that have been used before. AM-PAC 6 clicks offers one of many cross-disciplinary applications of instruments that can better predict and communicate patient outcomes.

**Funding for the Study:**

N/A